# The evolution of antimicrobial peptides in Chiroptera

**DOI:** 10.3389/fimmu.2023.1250229

**Published:** 2023-09-26

**Authors:** Francisco X. Castellanos, Diana Moreno-Santillán, Graham M. Hughes, Nicole S. Paulat, Nicolette Sipperly, Alexis M. Brown, Katherine R. Martin, Gregory M. Poterewicz, Marisa C. W. Lim, Amy L. Russell, Marianne S. Moore, Matthew G. Johnson, Angelique P. Corthals, David A. Ray, Liliana M. Dávalos

**Affiliations:** ^1^ Department of Biological Sciences, Texas Tech University, Lubbock, TX, United States; ^2^ Department of Integrative Biology, University of California, Berkeley, CA, United States; ^3^ School of Biology and Environmental Science, University College Dublin, Dublin, Ireland; ^4^ Department of Ecology and Evolution, Stony Brook University, Stony Brook, NY, United States; ^5^ Department of Biology, University of Central Florida, Orlando, FL, United States; ^6^ Department of Biology, Grand Valley State University, Allendale, MI, United States; ^7^ College of Science and Mathematics, University of the Virgin Islands, St. Thomas, VI, United States; ^8^ Department of Sciences, John Jay College of Criminal Justice, New York, NY, United States; ^9^ Consortium for Inter-Disciplinary Environmental Research, Stony Brook University, Stony Brook, NY, United States

**Keywords:** innate immunity, defensins, bioinformatics pipelines, non-model organisms, gene annotation, transposable elements

## Abstract

High viral tolerance coupled with an extraordinary regulation of the immune response makes bats a great model to study host-pathogen evolution. Although many immune-related gene gains and losses have been previously reported in bats, important gene families such as antimicrobial peptides (AMPs) remain understudied. We built an exhaustive bioinformatic pipeline targeting the major gene families of defensins and cathelicidins to explore AMP diversity and analyze their evolution and distribution across six bat families. A combination of manual and automated procedures identified 29 AMP families across queried species, with α-, β-defensins, and cathelicidins representing around 10% of AMP diversity. Gene duplications were inferred in both α-defensins, which were absent in five species, and three β-defensin gene subfamilies, but cathelicidins did not show significant shifts in gene family size and were absent in *Anoura caudifer* and the pteropodids. Based on lineage-specific gains and losses, we propose diet and diet-related microbiome evolution may determine the evolution of α- and β-defensins gene families and subfamilies. These results highlight the importance of building species-specific libraries for genome annotation in non-model organisms and shed light on possible drivers responsible for the rapid evolution of AMPs. By focusing on these understudied defenses, we provide a robust framework for explaining bat responses to pathogens.

## 1 Introduction

Host-pathogen interactions, dynamically influence the molecular, morphological, and structural traits of co-evolving organisms (1). Consequently, pathogens have undergone adaptations in their infectivity and virulence mechanisms in response to this evolutionary arms race, triggering an antagonist and immediate defense system by the host known as the innate immune response, which is an ancestral protection mechanism promptly activated upon pathogen invasion (2). This response involves the activation of organic molecules called antimicrobial peptides (AMPs), secreted by epithelial tissues in multiple copies and classes to effectively eradicate microbial agents (3).

AMPs are a group of small, cationic peptides, which serve as host-defense peptides (HDP) and possess an inherent cytotoxic activity, disrupting the cellular membrane of their targets, either by pore formation or changes in membrane permeability (4). The cytolytic activity of AMPs has been methodically demonstrated *in silico* (5), and *in vitro* against fungi, bacteria, and viruses (5–8). These features have recently drawn attention to the therapeutic application of AMPs (3, 5, 9–11). Their functions, however, are not constrained to exterminating non-self-agents but have also been associated with pleiotropic functions such as inflammation (10, 12), forming a bridge between the innate and adaptive immune responses (11).

AMPs are ubiquitous and have been described across the six life kingdoms (13). Their wide distribution is inherently accompanied by a remarkable structural diversity despite their small size, typically between 12 – 50 amino acids (14, 15). AMPs are more commonly known for being uniquely involved in immune functions (e.g., bacteriocin, cecropin, cytokine, histatin, Kunitz/bovine pancreatic trypsin inhibitor, lysozyme, thymosin; (14–17)). In multicellular eukaryotes, however, the formation of immune-like-peptides derived from proteolysis of larger molecules like histones or ubiquitins can play a crucial role in immunity (18–22). Nonetheless, defensins and cathelicidins comprise the AMP families that have been studied in several organisms (14) and are mainly secreted in epithelial tissues (23). Based on a highly conserved six-cysteine residue motif, which results in a differential number of disulfide bridges and, in consequence, a distinctive tertiary structure, defensins are classified into three subfamilies: alpha, beta, and theta (14, 24).

Given the importance of AMPs in immunity, selection to neutralize infections and grant different types of defense modulation by inducing pro- and anti-inflammatory effects (22) likely drives their expansion and diversification in vertebrates (23, 25–27). Given adaptation to differential microbial exposure and diverse ecologies, AMPs have a high predisposition for duplication and diversification (3, 28). High evolutionary rates resulting from duplication and diversification make inferring the evolution of vertebrate AMPs like defensins and cathelicidins challenging. Instead of having a broad action spectrum, AMP activity in host-defense mechanisms may be highly specific (3), resulting in adaptive turnover.

The variety of both defensins and cathelicidins has been shaped by gene duplication and subsequent selection (27, 29). To date, the molecular characterization of mammalian AMPs has been undertaken through next-generation sequencing of model organisms, or bioinformatic tools designed to target those organisms (e.g., Augustus). However, adaptive processes underlying these gene families remain overlooked in most non-model organisms. In one exception, in the monotreme *Ornithorhynchus anatinus* (29), duplication and functional diversification events shaped the evolution of defensin-like peptides and ultimately gave rise to venom components. Inferring the history of defensins may yield essential insights into the biology underlying gene family evolution but genome annotation using standard tools risks overlooking many of these short peptides.

Comprising more than 1,460 species (30), bats are a key group of non-model organisms. As the second largest group of mammals, the order Chiroptera is distinguished by unique adaptive traits such as their ability to sustain true flight (31). Their vast species diversity is reflected in their diversified feeding habits, the occupation of multiple ecological niches, and subsequent exposure to a wide range of pathogens including viruses known to cause mild to lethal pathologies in humans (e.g., coronaviruses, Hendra, Marburg, Nipah (32)), and have given rise to the development of an efficient and regulated immune response, allowing bats to have a high immune tolerance (32, 33). These unique adaptive traits make bats an ideal non-model organism to study host-defense coevolution.

An emerging consensus posits immune tolerance as a widespread evolutionary trait acquired by chiropterans, but most studies have focused on the adaptive immune response and its implications in the inflammatory response (33, 34). Despite its relevance in the defense against pathogens, studies pertaining to the evolution of the innate immune system and AMPs, specifically, are scarce (25, 35). AMPs are key actors in the innate response, yet rampant loss has been previously reported in bats (34). Our goal here is to assess how automated annotation influences the identification of these small genes. Specifically, we aim to determine whether reported AMP copy numbers and total duplications/losses constitute genuine evolutionary changes among species and to investigate the potential basis of their extraordinary AMP diversity and rapid evolutionary rates.

## 2 Materials and methods

Twenty high-quality and highly contiguous bat genome assemblies with >80% BUSCO completeness were selected to model the evolution of AMPs (**Table S1**). For ortholog identification and AMP prediction, we restricted our search to the major defensin and cathelicidin families. However, the targeted gene annotation performed in this study included all known and validated families of AMPs, see (13).

### 2.1 Ortholog identification in bat proteomes

To retrieve defensin and cathelicidin orthologs in bat proteomes (20 bat species across six Chiropteran families), we used protein sequences from 13 placental mammals (10 Boreoeutherians, two afrotherians, one xenarthran), and one marsupial species, allowing for the greatest diversity of AMP amino acid composition (**Table 1**).

**Table 1:** Mammalian AMP proteins used to identify orthologs in bat proteomes.

Species	α-defensin	β-defensin	Cathelicidin	Magnorder
*Bos taurus*	0	19	11	Boreoeutheria
*Canis lupus familiaris*	0	28	2	Boreoeutheria
*Ceratotherium simum simum*	1	17	3	Boreoeutheria
*Dasypus novemcintus*	19	16	3	Xenarthra
*Echinops telfairi*	1	12	1	Afrotheria
*Equus caballus*	23	21	2	Boreoeutheria
*Homo sapiens*	5	12	3	Boreoeutheria
*Monodelphis domestica*	0	1	13	Marsupialia*
*Mus musculus*	28	23	2	Boreoeutheria
*Octodon degus*	1	14	2	Boreoeutheria
*Orycteropus afer afer*	4	7	1	Afrotheria
*Otolemur garnettii*	1	18	1	Boreoeutheria
*Rattus norvegicus*	6	21	1	Boreoeutheria
*Sus scrofa*	0	21	9	Boreoeutheria
Total	89	230	54	

Taxonomic classification follows (36). The species of the Marsupialia infraclass is marked with an asterisk.

Amino acid sequences of α-, β-defensins, and cathelicidins of all non-bat species were downloaded from NCBI (37), and Ensembl (38); limiting the search to the Reference Sequence databases (**Table 1**). This dataset will be hereafter called the “*outgroup AMP dataset*”. For each of these three AMP classes, an initial amino acid multiple sequence alignment (MSA) was performed using default settings in MUSCLE v7.215 with 500 iterations (39). Each MSA was visually inspected in UGENE (40), and sequences were manually checked for duplicates, premature stop codons, and 4- or 6-cysteine residue motifs (cathelicidins and defensins, respectively). Sequences that did not meet these criteria were removed. For β-defensins, the MSA was separated into four sub-alignments because of the wide divergence and sequence dissimilarity among distantly related species.

Defensin and cathelicidins MSAs were used as input queries for an ortholog search using orthofisher (41). Orthofisher retrieves top ortholog hits and selects the ones meeting a percentage score based on the BUSCO pipeline criteria (42). We kept sequences with a bit score of >75% for the β-defensins, and >80% for the α-defensins and cathelicidins due to the differences in sequence conservation and divergence found in the AMP families of outgroup sequences. A final filtering step using SeqKit (43) retained sequences that contained at least two cysteine residues and no stop codons. The retrieved and filtered orthologs are hereafter referred to as the “*putative bat AMP dataset*”.

### 2.2 AMP gene prediction and functional annotation in bat genomes

The *putative bat AMP dataset* was subjected to an AMP probability test using both the caret v6.0.94 and ampir v1.1.0 R packages (44, 45). To avoid high false positive rates, the datasets used to train the model at this stage contained chiropteran proteins exclusively. Our model was trained with the positive and negative bat AMP datasets included in ampir, however, α-defensins and cathelicidins were added to the positive dataset to increase their representation in the local datasets. We downloaded these gene families from UniProt on July 10, 2022, searching the terms: *‘“cathelicidin”/”alpha defensin” AND (taxonomy_id:9397) AND (length:[1 TO 200])’*. Duplicates and sequences that lacked the 4-6-cysteine residue motif were discarded. Then, 21 random control proteins were downloaded using the query: “*Chiroptera [9397]”* to balance the number of positive and control sequences present in each dataset. We used a 70% threshold in our model to retain putative bat AMPs. Each AMP family was subject to a separate prediction and visual evaluation. However, to account for bat genome assembly misannotation and high gene family divergence, we retained all proteins, even if they lacked the 4-6 cysteine residue. The generated dataset will be herein referred to as “*predicted bat AMPs*”.

The targeted gene annotation of AMP genes followed the criteria of (5). Bat genome assemblies were soft-masked with RepeatMasker v4.1.2 using a curated library of mammalian Transposable Elements (TEs) described in (46). Genome assemblies were then annotated in a single round using MAKER2 v2.31.8 (47) and exonerate v2.2.0 (48). To match the size of AMPs, MAKER2 behavior options were changed in the default control files by setting a minimum contig length and an extended flank of 1,000 bp, the minimum required length of protein amino acids was set to 10, and extra steps to force start and stop codons were included.

The protein dataset used for homology inference within MAKER2 included 1) validated, predicted, and manually curated AMPs from bacteria, archaea, protists, fungi, plants, and animals downloaded from the 2020 release of the Antimicrobial Peptide Database (APD3; (13)); 2) the *outgroup AMP dataset*; and 3) the *predicted bat AMPs* dataset recovered with ampir. This concatenated dataset was then filtered for duplicates, non-standard amino acids, and sequences longer than 200 amino acids. The resulting protein dataset was composed of 3,694 sequences of species closely and distantly related to Chiroptera and predicted bat AMPs.

To improve the prediction of the precursor region of the genes, we included cDNA sequences downloaded from NCBI on July 31, 2022, as Expressed Sequence Tags (ESTs) evidence by searching the phrase: “*((antimicrobial) AND precursor) AND Mammalia”*, including sequences up to 10,000 bp. The 417 cDNAs were then aligned to the bat genomes using GMAP (49) with the parameters suggested by (5): -A –max-intronlength-ends = 200000 -O -n20 –nofails.

Filtering the results of MAKER2 annotation consisted of keeping protein sequences that had a length between 10 and 200 amino acids, used standard amino acids, and lacked premature stop codons. The functional annotation of the resulting proteins was performed with InterProScan 5 (50). The results of the search were then separated into a dataset containing α-, β-defensins, and cathelicidins and a second dataset with any other putative AMP protein. Both datasets were then separately subjected to a final round of AMP probability prediction with ampir.

For the dataset containing all proteins but defensins and cathelicidins, we used the built-in “ampir_mature” model of ampir and set a 70% threshold. This model was chosen because AMP annotation in MAKER2 was not targeted for the precursor region of other AMP families, and this model reduces the number of false positives (44). Moreover, the resulting predicted proteins were checked against the list of names of APD3, and literature mentioning putative functions of cryptic AMPs (22, 51).

For the defensin and cathelicidins dataset, we trained a *de novo* model with caret and ampir using positive and negative training sets following (5), and used an 80% threshold to keep positive sequences. The positive “*potential AMP dataset”* was subset to contain only defensins and cathelicidins of lengths ≥ 10 amino acids. Peptide length distributions in the positive and negative datasets were then approximated as recommended by (5) with a custom R script. The proteins resulting from the prediction were then visually examined and sequences that lacked the 4 or 6 cysteine motifs were discarded. Ultimately, to identify and annotate AMP families and subfamilies of the α-, β-defensins, and cathelicidins, we blasted the proteins predicted by ampir against the *potential AMP dataset* using the command line BLASTP (52), and only the best hit with an e-value < 1e-6 was retained. This exhaustively curated set of defensins and cathelicidins proteins of bats was used in all downstream analyses. A summary of this pipeline is presented in **Figure S1**.

### 2.3 Gene structure and genomic organization

Custom bash scripts were used to estimate gene length, exon number, and to retain sequences with nucleotide lengths ≥ 200 bp as smaller gene lengths did not translate for full peptide sequences (i.e., signal, prepropeptide, and mature regions). Filtering based on number of exons (≥ 2) was not used since we detected that single-exon β-defensin genes coded for full-length AMPs. The genomic organization of these genes was inferred by visually comparing the composition of gene clusters with the gggenes R package (53).

To assess the presence of TEs within genes, we used the intersect BEDTools function (54) with the coordinates of defensin and cathelicidin genes against TEs bed files obtained with the RM2bed.py script (https://github.com/davidaray/bioinfotools). To reduce potential biases of TE counts due to masking errors, we overlapped TEs that were as close as 10 bp on the same DNA strand, and that reciprocally covered at least 80% of the longest overlapping TE. When the automatic resolution of the overlap was not possible, we kept the TE with the lowest divergence value to the consensus. Repeats characterized as simple or low complexity were discarded.

Initial inspection of the TE content in AMP genes suggested longer genes had more TE content. To estimate the relationship between gene length and TE content, we applied Bayesian hierarchical models. A Bayesian hierarchical framework enables including both sample-wide and group-specific effects with the latter accounting for correlations among observations from the same species and other group-specific effects such as the AMP gene family. After summarizing the total TE length and number of TEs per AMP gene (**Table S2**), models were fitted using the R package MCMCglmm (55). To account for the phylogenetic relatedness among species, we included a species-specific effect in the model whose initial values were given by the inverse relatedness matrix specified by the phylogeny. Total TE length, however, is not expected to be the only influence on gene length, and both the number of exons and count of TEs were included as covariates. Both gene length and TE length were log-transformed, and models ran for 1,000,000 iterations with a burn-in of 1,000 and thinning every 500 iterations.

### 2.4 Orthology inference

We utilized OrthoFinder v2.5.4 (56) to infer phylogenetic Hierarchical Orthogroups (HOGs, see https://github.com/davidemms/OrthoFinder), incorporating the laurasiatherian species *Bos taurus*, *Canis lupus familiaris*, *Equus caballus*, and *Sus scrofa*, whose peptides were extracted from the *outgroup AMP dataset*. To determine the HOGs, we employed gene tree inference using a MSA obtained *a priori* with Clustal Omega by running a maximum number of 100 guide tree and Hidden Markov Model (HMM) iterations in UGene (57), a default inflation parameter of 1.5 for Markov clustering (MCL) of proteins (58), and a rooted ultrametric species tree. This strategy was used to alleviate any potential biases arising from differential rates of sequence evolution, thus, increasing accuracy.

The protein-coding sequences (CDs) available in (34) annotated using MAKER2 were combined with the aforementioned laurasiatherian species and used to infer an ultrametric tree. Only sequences with an annotation edit distance (AED) ≤ 0.2 and a minimum of 20 species were kept. These genes were aligned with MACSE (59) to maintain the correct open reading frame and were used to infer Maximum Likelihood (ML) gene trees using IQ-TREE 2 (60). This dataset comprised 1,358 genes concatenated into a supermatrix. Using the best-fit models of sequence evolution per gene, the supermatrix was used to infer the species tree with IQ-TREE 2. In the initial species tree, Carnivora (*Canis lupus* familiaris) and Cetartiodactyla were sister taxa, with Perissodactyla sister to non-bat mammals. To be consistent with previous mammalian topologies (31, 34), *C. l. familiaris* was constrained as sister to Perissodactyla+Cetartiodactyla and branch lengths were recalculated. Divergence times for the ancestral nodes of Cetartiodactyla, Chiroptera, Yangochiroptera, and Molossidae+Vespertilionidae were constrained using fossil calibrations, and ultrametric tree branch lengths were inferred using penalized likelihood in r8s v1.81 (61).

### 2.5 Gene family evolution

To infer changes in gene family size and rates of evolution of the α-defensin, β-defensin, and cathelicidin gene families, we used the HOGs inferred by OrthoFinder and the developer version of CAFE 5 (62). For this, we considered two different birth-death models: 1) Among gene family variation with a discrete gamma model with three categories K = 2, 3, and 5; 2) a multi-λ model with two different birth-death parameters, λ = 3 and 5 assigned to different parent and child clades in the ultrametric tree. Both models included default settings and an error parameter to account for genome assembly errors. Each category within the models was run 30 times to check for convergence of the Model Final Likelihood (-lnL). The between-model comparison was made by selecting the highest lnL, and a likelihood ratio test (LRT) with the R v4.2.1 base package was used to compare the goodness of fit. The more complex model was judged as a better fit when LRT yielded a p-value < 0.05 compared to a chi-squared distribution with degrees of freedom equal to the difference between the number of parameters of the models compared.

### 2.6 Evolutionary histories of AMPs

In contrast to other vertebrates and mammals, the evolutionary history of defensins and cathelicidins has not been explored in bats, hence we inferred speciation and duplication events by gathering comprehensive evidence from OrthoFinder, and CAFE 5. OrthoFinder automatically reconciled all gene trees with the ultrametric species tree via duplication-loss-coalescence (DLC) to determine duplications with a species overlap method (56). This pipeline considers the existence of a duplication event if at least 50% of the child clades have retained both duplicated genes. Only HOGs inferred to have experienced duplications were selected for comparison with CAFE 5.

### 2.7 Validation of gene expression in transcriptomes

Transcriptome assemblies were generated for 15 bat species using a combination of published RNAseq data from liver, intestine, and kidney samples, and newly generated data from lung samples (**Table S3**). First, we assessed the quality of the transcripts on all read files with FastQC (63) and trimmed the adapters from the reads with fastp (64). Then, we generated assemblies using the Oyster River Protocol, hereafter ORP (65), which combines four different assemblers, assesses the outputs of those assemblers, and combines the results based on a quality threshold to produce an optimal *de novo* transcriptome assembly.

We applied dammit! (https://github.com/camillescott/dammit) to annotate our ORP assemblies and determine the correspondence between transcripts and genes. Redundant transcripts were identified and excluded from the ORP assemblies. To perform the annotation with dammit!, we employed the pep files generated from the transdecoder analysis (https://github.com/TransDecoder), which contained protein sequences obtained through blasting and the associated transcripts from the ORP assemblies. Using VSEARCH (66), we clustered these transcripts from the transdecoder output for each tissue in each species, selecting a representative transcript for each gene based on 95% identity in amino acid sequence. This representative transcript is referred to as “the transcript” in our analysis.

To validate our curated database, we interrogated the annotated transcriptomes using tblastn v.2.7.1+ (52), and only considered true transcripts those with an identity score greater than 80%. Finally, we manually validated the transcripts by aligning them against our curated database, using Geneious prime v.2023.1.2 (https://www.geneious.com).

## 3 Results

### 3.1 AMP diversity

**Figure 1:**
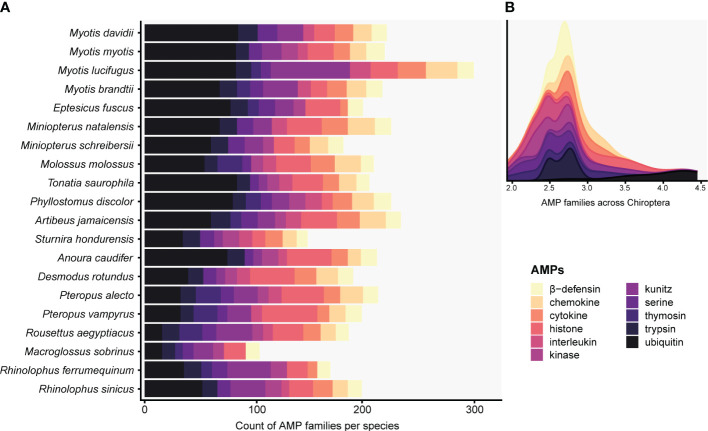
Histogram **(A)** and stacked density **(B)** plots display predicted AMPs present in at least five Chiropteran species with counts greater than seven. The total counts of the targeted defensins and cathelicidins families can be seen in **Table S4**. The x-axis in B is expressed as a logarithmic scale.

The targeted genome annotation of 20 bat genome assemblies recovered 29 AMP families (**Table S4**). Out of the 4,143 total annotated proteins, only 1,162 (28.04%) were predicted to be AMPs. Conventional and cryptic AMPs were part of the 829 proteins detected by the ampir built-in “predict_mature” model and encompassed 90.17% of AMP diversity. These putative AMPs, mostly present as multiple copies in all species, were comprised of chemokine, cytokine, histone (e.g., H2A, H2B, H3/CENP-A), interleukin, kinase, kunitz, serine, trypsin, and ubiquitin (**Figure 1A**; **Table S4**). In fact, ubiquitin, kunitz, histones and other cryptic AMPs contributing the most to total diversity in Chiroptera and had counts ≥ 7 copies per species (**Figure 1B**). In contrast, AMPs *sensu stricto* were retrieved in low counts or as single copy genes: amyloid, eotaxin, lysozyme, resistin, saposin, and WAP (**Table S4**). Furthermore, hepcidin was detected in the genus *Myotis* except for *M. myotis*. Adrenomedullin was found in multiple copies solely in *Anoura caudifer* and *Myotis brandtii*, whereas thymosin was absent in *A. caudifer* and *Myotis davidii*. Angiogenin and elafin were restricted to *M. myotis* as single copies (**Table S4**).

Although several other AMPs were predicted by ampir, we discarded those proteins since they were not present in the APD3 database, and the validation, verification, and description of the wide array of potential new AMP families discovered is beyond the scope of this work.

**Figure 2:**
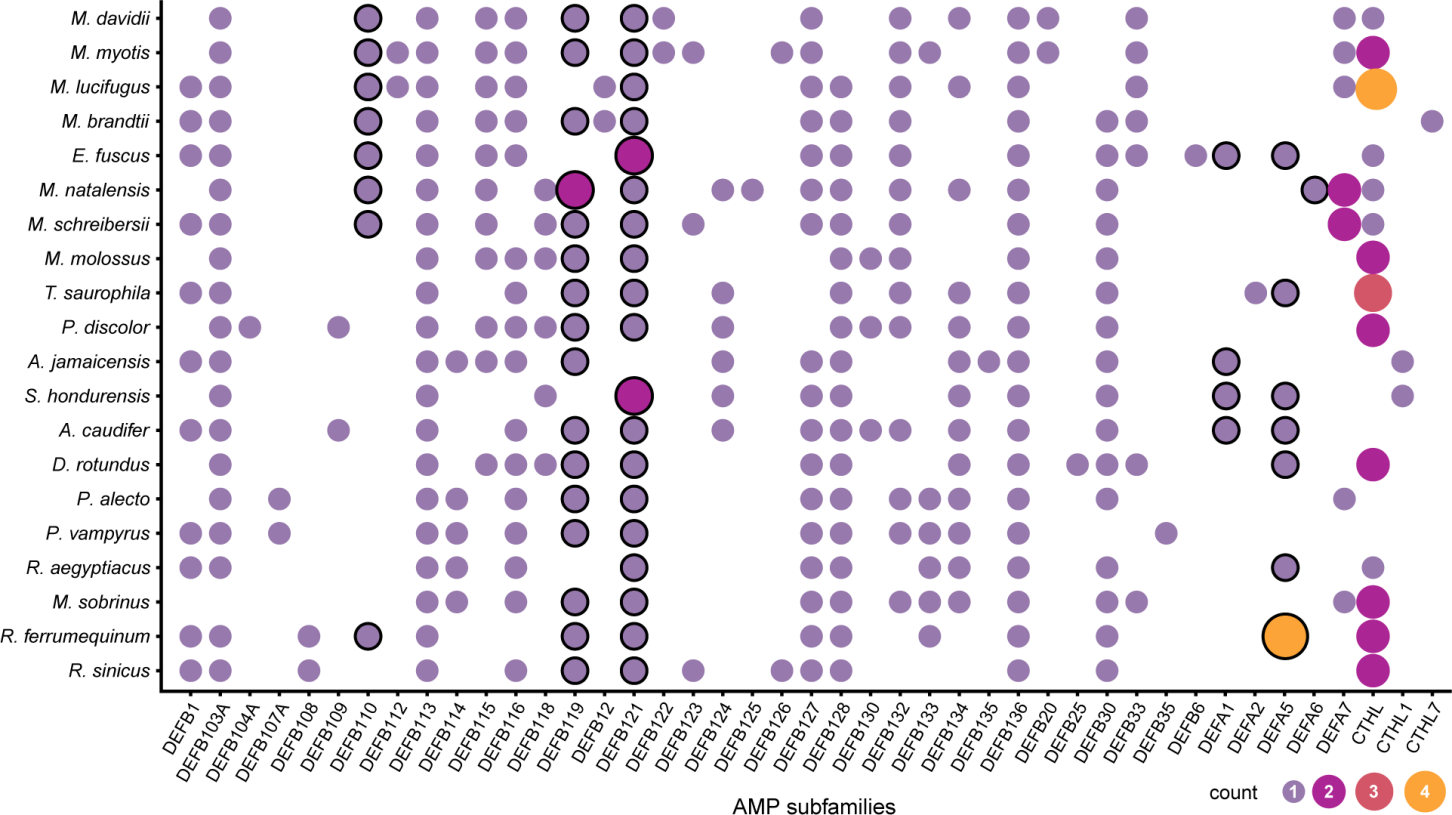
Distribution of putative defensin and cathelicidins subfamilies in Chiroptera. The size and color of the circles represent the number of genes per subfamily. The circles with a black stroke are subfamilies previously reported as absent in Chiroptera.

The predicted α-defensins, β-defensins, and cathelicidins consisted of 333 proteins which accounted for 9.83% of AMP diversity and were detected by our *de novo* trained model with excellent performance (**Table 2**). The distribution and proportion of AMPs among bats were uneven (**Figure 2**). α-defensins were absent in *Myotis brandtii*, *Molossus molossus*, *Phyllostomus discolor*, *Pteropus vampyrus* and *Rhinolophus sinicus.* Likewise, cathelicidins were not recovered in *Anoura caudifer*, *Pteropus alecto* and *P. vampyrus* (**Figure 2**). Species for which α-defensins were retrieved possessed at least one copy of the DEFA1, DEFA5, and DEFA6 subfamilies. Only a single copy of the mature peptide of the DEFA2 subfamily was found in *Tonatia saurophila* (**Figure 2**). *Rhinolophus ferrumequinum* harbored four copies of α-defensins located ~6 Kb from each other, two of which were exact duplicates (see below). Likewise, a cluster of three α-defensins was observed in *Miniopterus natalensis*.

**Table 2:** *De novo* trained model performance for the detection of α-, β-defensins, and cathelicidins.

Parameters	Percentage
Sensitivity	94.32
Specificity	91.30
Positive prediction value	91.52
Negative prediction value	94.17
Precision	91.52
Recall	94.32
F1	92.90
Prevalence	49.89
Detection rate	47.05
Detection prevalence	51.41
Balanced accuracy	92.81

The β-defensins had the greatest diversity and were present as single-copy genes in all species, except for the subfamilies DEFB119 in *Miniopterus natalensis*, and DEFB121 in *Eptesicus fuscus*, and *Sturnira hondurensis* (**Figure 2**). Nevertheless, β-defensin subfamilies were differentially distributed. The subfamilies DEFB113 and DEFB136 were found in all species, whereas DEFB123 and DEFB130 were found only in three species, and DEFB108, DEFB109, DEFB112, DEFB12, DEFB122, DEFB135, DEFB20, and DEFB6 were each in fewer than 3 species (**Figure 2**). Gene clusters were formed of different subfamilies and comprised 3 – 4 genes within a 2 – 10 Kb extent in some scaffolds, with one α-defensin gene rarely found in the vicinity of the β-defensin clusters (**Table S5**). In contrast, cathelicidins were retrieved as multi-copy genes, with at least two copies present in most species (**Figure 2**), and only two genes in *Myotis lucifugus* were grouped as close as ~8 Kb (**Table S5**).

Expression of validated α- and β-defensin genes in tissue samples was predominantly found in the intestine across all species, while the lung exhibited low expression of β-defensins (**Figure S2**). Cathelicidins were consistently observed in all analyzed tissues, with similar expression levels in the intestine and lung (**Figure S2**). Furthermore, we examined the expression of these genes based on dietary habits and noted that α-, and more abundantly, β-defensins were highly expressed in insectivores. Cathelicidins were distributed evenly among frugivores and insectivores, and the sanguivore *Desmodus rotundus* exhibited the third highest expression abundance (**Figure S2**; **Table S3**). Carnivores and nectarivores displayed the lowest expression of these AMPs (**Figure S2**).

### 3.2 Gene structure changes are driven by differential TE accumulation

**Figure 3:**
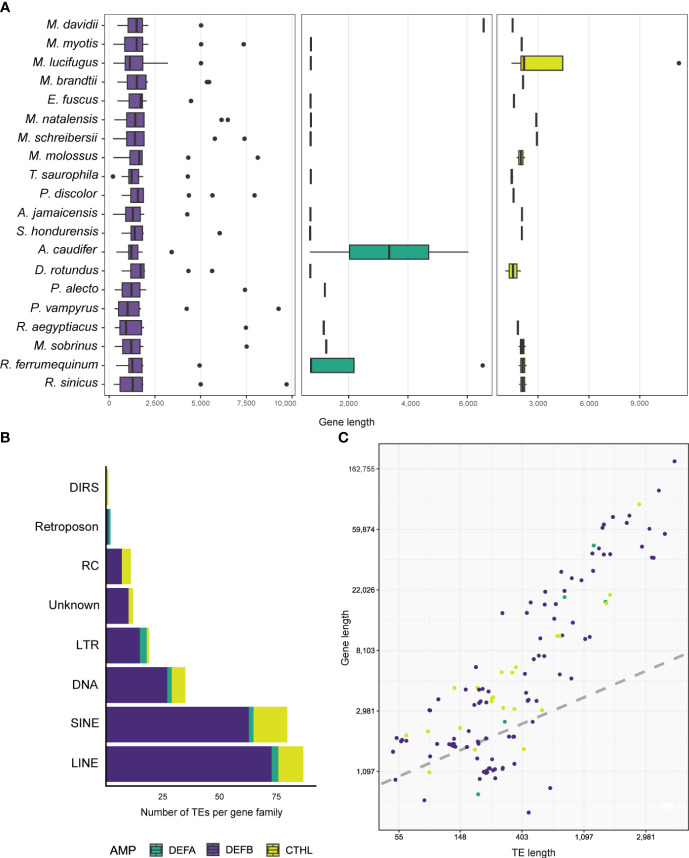
Boxplots depicting the distribution of gene lengths in different AMPs **(A)**. Stacked barplot shows the number and types of TEs accumulated in the introns of AMPs **(B)**, and linear regression showing the relationship between gene and TE lengths **(C)**.

Genes that translated into full defensin and cathelicidin amino acid sequences ranged between 206 – 11,306 bp, and all species were found to have at least one gene greater than 2,500 bp (**Table S5**). The greatest length variability was found in the β-defensins with a median of 1,421 bp (**Figure 3A**; **Table 3**), whereas α-defensins and cathelicidin gene lengths were similar, with medians of 728 and 1,990 bp respectively (**Figure 3A**; **Table 3**).

**Table 3:** The number of genes that code for full-length AMP peptides.

Exons	α-defensin (25)	β-defensin (234)	Cathelicidin (27)
1	–	30 (13%)	–
2	22 (88%)	199 (85%)	1 (3.7%)
3	3 (12%)	5 (2.1%)	4 (15%)
4	–	–	22 (81%)
Gene length	728 (699 – 6,552)	1,421 (206 – 9,702)	1,990 (1,080 – 11,306)

The percentage of single and multi-exon AMP genes in Chiroptera is shown in parentheses. Median gene lengths are displayed with minimum and maximum values.

Antimicrobial peptides appear to be translated from single and multi-exon genes. We annotated and characterized 22 two-exon (called enteric) and 3 three-exon (called myeloid) α-defensin genes respectively (**Table 3**). Of the 234 β-defensins, thirty genes were organized as single-exon, 199 as two-exon, and 5 as three-exon genes (**Table 3**). Cathelicidins were mostly coded from 22 four-exon genes, although 4 three-exon genes and a unique two-exon gene in *Eptesicus fuscus* were also annotated (**Table 3**). The latter is a single-copy gene with an insertion of a 414 bp Ves SINE (Short Interspersed Nuclear Element), a unique kind of bat TE (**Table S2**), and codes for a protein with a relatively large amino acid insertion in the cathelin propeptide (see below).

Five α-defensins of five different species were detected to have a TE insertion (**Table 4**), including one of the four genes present in *Rhinolophus ferrumequinum* (**Table S2**). Approximately 50% of the β-defensins, and 80% of the cathelicidin genes contained several TE types embedded in their introns (**Tables 3**, **4**). Long Interspersed Nuclear Elements (LINE) and SINEs were retrieved in all species, whereas a unique Dictyostelium Intermediate Repeat Sequence (DIRS) was found in one cathelicidin gene of *Miniopterus schreibersii*, and two retroposons in one α and β- defensin of *Myotis davidii* and *Molossus molossus*, respectively (**Figure 3B**, **Tables 4**, **S2**).

**Table 4:** Number of genes that contain TEs in their introns.

TE type	α-defensin (5)	β-defensin (107)	Cathelicidin (22)
DIRS	–	–	1 (1.6%)
DNA	2 (13%)	40 (12%)	6 (9.7%)
LINE	6 (38%)	156 (47%)	20 (32%)
LTR	5 (31%)	21 (6.3%)	3 (4.8%)
RC	–	12 (3.6%)	5 (8.1%)
Retroposon	1 (6.3%)	1 (0.3%)	–
SINE	2 (13%)	94 (28%)	24 (39%)
Unknown	–	11 (3.3%)	3 (4.8%)
TE length	188 (111 – 732)	177 (11 – 2,677)	148 (52 – 1,597)

The representation of each TE type in each AMP is shown as a percentage. The median length of the TEs is shown with minimum and maximum values in parentheses.

Members of the Vespertilionidae, Miniopteridae, and Molossidae displayed a similar composition and wide diversity of TEs, including Long Terminal Repeats (LTR), Rolling Circle (RC), and unknown TEs (**Figure S3**). Likewise, the rhinolophids portrayed a similar composition of TEs although in lower copies than their strict insectivore counterparts (**Figure S3**). The Phyllostomidae and Pteropodidae families showed the least diversity and copy number of TEs, except for *Phyllostomus discolor* and *Desmodus rotundus* (**Figure S3**; **Table S2**). In fact, the genes of the DEFB116 and DEFB128 subfamilies in *Pteropus alecto* and *P. vampyrus* contained SINEs and LINEs exclusively (**Figure S3**; **Table S2**).

Longer genes had more TEs. For example, one DEFB123 gene in *Rhinolophus sinicus* was 9,702 bp long (**Figure 3A**) and accumulated a total of 19 TEs representing LINEs, SINE, DNA, LTRs, and other TEs, composing almost half of the gene length (**Table S2**). A similar number and diversity of TEs was found in the longest α-defensin (1,284 bp of the introns) and cathelicidin (2,682 bp of the introns) genes (**Table S2**).

Results from Bayesian hierarchical models confirmed a positive association between TE length and AMP gene length (**Figure 3C**). After controlling for gene length and TE count, the coefficient of TE length on gene length was positive and sample-wide parameters (sometimes called ‘fixed’ effects) explained almost 60% of the variance in gene length (**Table 5**). This positive relationship between length does not arise through sheer TE numbers, as shown by the negative coefficient of number of TEs on AMP gene length (**Table 5**). Species-specific phylogenetic effects are nevertheless important, as shown by the difference between the overall fitted line and the observations (**Figure 3C**). These differences are not captured by different intercepts for AMP-gene-subfamilies, whose 95% high probability density overlapped with zero (**Table 5**).

**Table 5:** Parameter estimates from hierarchical Bayesian models of AMP gene lengths as a function of TE length, with exon and TE counts as covariates.

Type of parameter	Parameter	Mean	Lower HPD 95%	Upper HPD 95%	ESS
Sample-wide	Intercept	5.083	3.747	6.556	2334
Sample-wide	Exon count	0.120	0.098	0.144	1998
Sample-wide	(log) TE length	0.436	0.290	0.588	1998
Sample-wide	TE count	-0.030	-0.062	0.007	1998
AMP-specific	DEFA intercept	0.292	-0.882	1.497	2164
AMP-specific	DEFB intercept	0.138	-1.061	1.262	2225
AMP-specific	CTHL intercept	-0.359	-1.631	0.764	2202

Both gene and TE lengths were log-transformed. ESS = effective sample size, HPD = high probability density. The *R^2^* of the model for sample wide effects was 0.59 (95% HPD 0.27, 0.80).

### 3.3 Gene family evolution

Out of 469 defensin and cathelicidin genes that included both bats and outgroup species, 37 HOGs were identified by OrthoFinder with a median of 14 genes per HOG (**Table S6**). All α-defensins, except one in *Tonatia saurophila*, were assigned to a single HOG, whereas the cathelicidins were split into two HOGs (**Table S7**). The β-defensins were assigned to 34 HOGs, with at least two orthologs each (**Tables S6**, **S7**).

**Figure 4:**
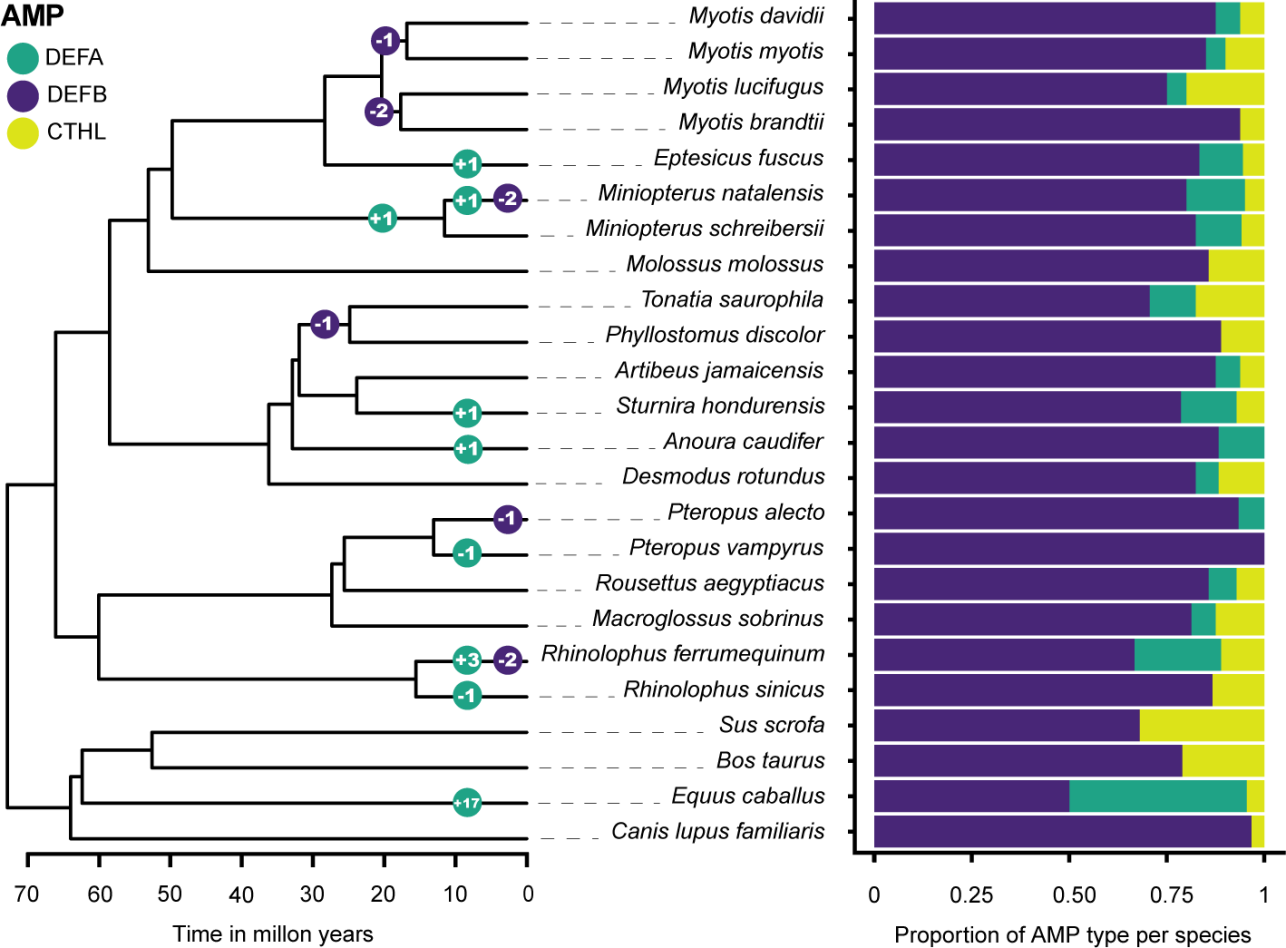
Gene family size changes in the evolutionary history of six Chiropteran families. The color-coded circles in nodes and branches of the ultrametric tree display the number of significant expansions and contractions of gene subfamilies. β-defensin expansions are shown as a total and detailed in **Table S8**. The proportion of each subfamily per species is shown in a stacked bar plot.

Gene family size changes inferred in CAFE 5 compared the k=3 discrete gamma (lnL = -648.749) model and the λ = 5 models (lnL = -627.106). The more complex model was a significantly better fit (p = 3.98e-10). The HOGs composed of α-defensins, and the β-defensin subfamilies DEFB1, DEFB114, DEFB123, and DEFB126 were statistically significant p < 0.05 in some branches and nodes (**Figure 4**; **Table S8**).

The overall change in family sizes was greater in Vespertilionidae+Miniopteridae, followed by Rhinolophidae, Phyllostomidae, and Pteropodidae (**Figure 4**). Significant expansions of the α-defensins were inferred in *Eptesicus fuscus, Miniopterus natalensis, Sturnira hondurensis, Anoura caudifer*, *Rhinolophus ferrumequinum*, and in the most recent common ancestor of Miniopteridae (**Figure 4**). Conversely, contractions with complete loss of the α-defensins were inferred in *Pteropus vampyrus* and *Rhinolophus sinicus* (**Figure 4**).

Regarding the β-defensins, contractions with subsequent gene losses were inferred for DEFB1 in the ancestral node of *Myotis myotis* and *M. davidii*, two gene losses of DEFB123 and DEFB126 in the ancestor of *Myotis lucifugus* and *M. brandtii*, and one contraction of DEFB114 in the ancestor of *Tonatia saurophila* and *Phyllostomus discolor* (**Figures 2**, **4**; **Table S8**). Furthermore, losses of DEFB1 and DEFB123 were predicted in *Miniopterus natalensis*, DEFB1 in *Pteropus alecto*, and DEFB123 and DEFB126 in *Rhinolophus ferrumequinum* (**Figure 4**).

**Figure 5:**
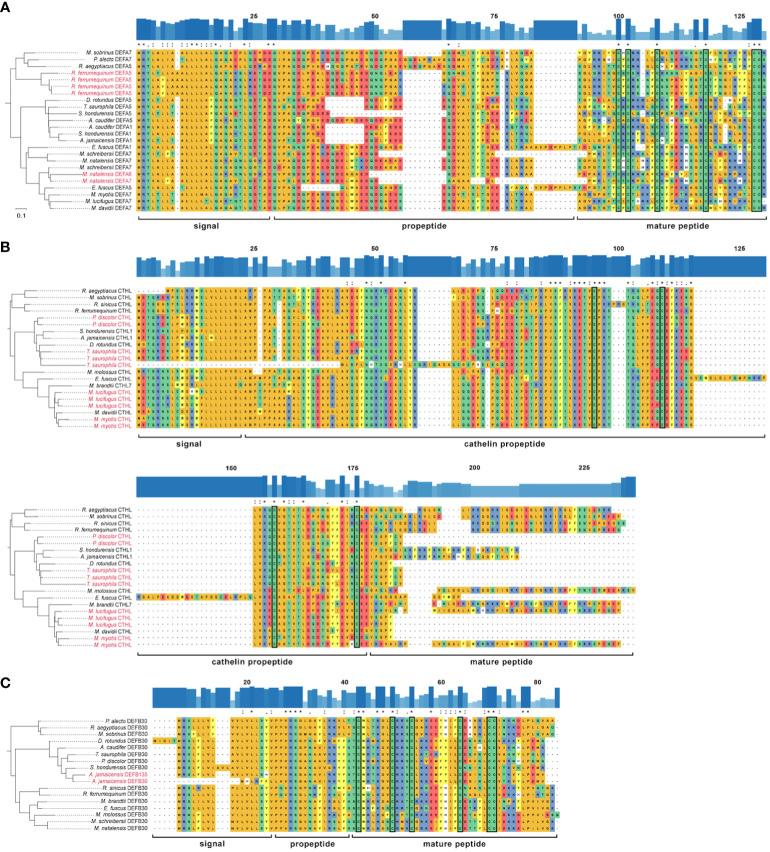
Rooted reconciled amino acid gene trees and multiple sequence alignment of three HOGs: **(A)** α-defensin; **(B)** cathelicidin; **(C)** β-defensin DEFB30 subfamily. Outgroups are not shown. Amino acids are colored according to their side-chain chemistry. Significant duplication events inferred by OrthoFinder are colored red on the labels of terminal branches. The degree of conservation of the sequences is shown above each alignment as bar plots and identical positions are marked with an asterisk, highly similar with a colon, and with a moderate identity with a period. The conserved cysteine residues are enclosed in a rectangle.

OrthoFinder, in contrast, predicted significant duplication events of 12 HOGs in the terminal branches of all outgroup species (**Table S9**). For the ingroup, duplication events were predicted only for the HOG composed of α-defensins in the terminal branches of *M. natalensis* and *R. ferrumequinum* (**Figure 5A**); one HOG composed of cathelicidins in the terminal branches of *M. myotis*, *M. lucifugus*, *T. saurophila*, and *P. discolor* (**Figure 5B**) and the HOG composed of the DEFB30 subfamily in the branches of *A. jamaicensis* (**Figure 5C**). The latter included a sequence functionally annotated as DEFB135 of *A. jamaicensis*.

### 3.4 Sequence diversity

All the α-defensins were annotated as full length, i.e., signal, propeptide and mature regions (**Figure 5A**). α-defensins were the shortest peptides, ranging between 93 – 119 aa, and its length variability was mostly caused by deletions in the propeptide region in four phyllostomid, and one vespertilionid species, and insertions in the propeptides of two pteropodid and one vespertilionid species (**Figure 5A**).

Cathelicidins were the longest peptides (134 – 181 aa) and they were highly similar across their signal and propeptide regions. The mature peptides presented a remarkable diversity and 10 of them were partially recovered. Moreover, a highly conserved 59 aa region towards the signal peptide was also retrieved in *Myotis myotis, P. discolor*, and *T. saurophila* (**Supplementary Data**), although this conserved region was trimmed in the MSAs because we could not identify it in any other bat species. The propeptide made up more than half of the cathelicidin lengths, and it was larger in *Eptesicus fuscus* due to a 39 aa insertion (**Figure 5B**).

The annotation of β-defensins proved to be intricate and only the propeptide and mature regions could be annotated for 233 peptides from a total of 270 β-defensins (**Supplementary Data**). AMP lengths were highly variable, even within gene subfamilies, and spanned 60 – 147 aa. The signal and proregions were highly conserved in all subfamilies and only a few amino acid mutations resulted in a change of their side-chain chemistry (**Figure 5C**), unlike the α-defensins, which showed a higher degree of variability in the signal and proregions.

## 4 Discussion

As both direct inhibitors of diverse pathogens and effectors of the innate immune system, AMPs can be the first line of immune defense, mediating relationships between hosts and microbiomes across epithelia. In bats, previous genome annotations reported the putative loss of certain defensins with the potential to modify immune responses against viruses and other pathogens (34). However, exhaustive annotation of AMPs in high-quality genome assemblies reveals variability in AMP repertoire within bats beyond the simple model of ancestral loss first proposed, including significant lineage-specific expansions and contractions.

By complementing in-depth annotation with machine learning for AMP probability prediction, we built a curated bat-specific database of antimicrobial peptides and retrieved the three targeted gene families —α-, β-defensins, and cathelicidins— corroborating the loss of some of these genes and recovering AMPs that were previously considered lost in bats because of the limitations of standard annotation methods (**Figure 2**). Some previously reported losses were annotation artifacts, probably caused by the high content of TEs within introns of varying lengths, undermining the inference of evolutionary changes in AMP copy numbers and total duplications/losses. These improved AMP annotations are therefore central to explaining relationships between bat species and the diverse viruses, bacteria, fungi, and parasites they host.

By estimating changes in gene family sizes, we discovered expansions and contractions of the α-defensin gene subfamily, which was previously believed to be lost in bats. These gains and losses were mostly concentrated in the families Miniopteridae and Rhinolophidae. Based on our transcriptomic data, and evolutionary analyses we hypothesize that a possible higher exposure to viruses and bacteria because of their diet (67), contributes to the pattern dynamic α-defensin birth death seen here, and explicitly shown in gene expression differences of a proportion of these repertoires in selected species of varying feeding habits (**Figure S2**). Nevertheless, further validation through tissue-specific RT-PCR can help localize expression and overcome the limitations of bioinformatic analyses.

While pteropodids and phyllostomids also display different rates of gains and losses of α-defensins (**Figure 5**), insectivory is less likely to play a role in their dynamics. Phyllostomids comprise the most ecologically diverse clade in Chiroptera, with the widest range of diets in all bats, and indeed, all mammals. Although ancestral phyllostomids are inferred to be insectivorous, descendent lineages include carnivores, exclusive blood feeders such as *Desmodus rotundus*, nectarivores, palynivores, and predominantly frugivores and the rapidly diversifying Stenodermatinae subfamily (68). This dietary plasticity is associated with differential composition of gut microbiota as emphasized by (69), and we propose a further link to the presence or absence of α-defensins. Both α-defensins and cathelicidins are secreted in gastrointestinal epithelia (14), specifically DEFA5 and DEFA6 expression is exclusive to the Paneth cells (70), and thus likely play an important role in the maintenance of gut microbiota (70, 71). Gut microbiota, in turn, are known to modulate the immune system of the host (72, 73), hence α-defensin variation could potentially be driven in part by diet-related microbiome evolution, and we hypothesize a similar trend in cathelicidins despite no significant inferred contractions/expansions by CAFE.

In contrast to α-defensins and cathelicidins, no statistically significant shifts were found in most β-defensin subfamilies. One factor driving this pattern is the high diversity of these genes, which is reflected by their predominance across all lineages studied and possibly enhanced by the abundant presence of intronic TEs that might perturb transcription (74, 75), promote gene duplication and modify chromatin accessibility when present around the genes (76, 77). Accumulation patterns for specific TE types resemble those reported from whole bat genomes (31), suggesting lineage-specific TE insertions in AMP introns reflect the overall insertion dynamics for the genome and not particular to these genes. The lack of significant gains/losses in β-defensins, however, may also indicate that the specific function of the β-defensin subfamilies has been highly conserved in the evolutionary history of Chiroptera. β-defensins are expressed in mucus-producing tissues, which are constantly exposed to pathogens (73), and some β-defensins are known to control bacterial populations and homeostasis in the digestive tract (78). These defensins operate by activating specific host defense mechanisms (3), either by attacking the pathogen itself or by modulating the immune response, downregulating receptors, and ligands such as CD4, CCR6, CCXR4, and CXCL5 (11). Given their anti-microbial and immune effector functions, negative selection against loss or duplication may be strong enough to maintain most subfamilies through time (79). Functional importance would therefore explain the pattern of duplications and losses becoming rare in these subfamilies, despite their high diversity.

Our analyses reveal unique α- and β-defensin losses in yinpterochiropteran bats that could be associated with their interactions with viral pathogens. *Pteropus vampyrus* and *Rhinolophus sinicus* lack α-defensins, and *P. alecto* has only one copy and experienced one significant contraction of the β-defensin DEFB1. This is unlike *R. ferrumequinum*, whose α-defensin genes have originated through tandem duplications (**Figures 2**, **5**). Variation among these species is important since *P. alecto* is the known reservoir of Hendra virus (80), *Rhinolophus sinicus* has been found to harbor SARS-like viruses (81, 82), and both alpha- and beta-coronaviruses circulate in *P. vampyrus* (82). While rhinolophids are strict insectivores, the pteropodids sampled are, like many phyllostomid lineages, exclusively frugivorous. We hypothesize these clade-specific losses have been positively selected due to the advantageous maintenance of microbiota of specific dietary habits and could play a role in viral tolerance as initially hypothesized by (34).

Enhanced annotation of existing genome assemblies is the foundation of AMP identification and analyses. While tools that rely on using reference genomes (e.g., human and mouse) can successfully annotate a substantial portion of protein-coding genes (80 – 98%), standard annotation pipelines may still overlook or misidentify duplicated or highly divergent genes in non-model organisms. Thus, there is an increasing need to supplement genome annotation with functional annotation and machine learning software specifically trained to retrieve and validate genes of interest *in silico*.

Investigating bat immunity cannot solely rely on automated tools. As with AMPs, such tools may lead to incorrect inference of gene gain/loss because their high mutation rates, large-scale evolutionary changes, and size render them invisible to standard tools. Discovering species-specific, divergent genes in clades with a long evolutionary history as bats (~ 65mya; (83)) requires custom approaches because of the adaptive character of these gene families. Adaptive immune gene losses have been proposed, specifically among gene families involved in the activation of the inflammasome (31, 34, 84). Some defensins were previously reported as lost in bats (34) but our detailed examination revealed their presence in some bat genomes (**Figure 2**).

Our analyses also reveal extensive within-Chiroptera variation across both the three main AMP families and many other gene families besides, but interpreting the biological meaning of these changes will require future functional analyses. The wide diversity of AMPs retrieved, which some researchers consider cryptic (22) like the peptides derived from histones and ubiquitin, have been described to have antimicrobial, antibacterial, and antifungal immune roles in frogs, scallops, and prokaryotes (19–21, 85), and histones specifically are known for being immune modulators by acting as damage associated molecular patterns (DAMPS; (18)). What roles might histones, ubiquitins, and other HDPs play in bats, and are they more involved in the immune response than previously thought? Could selection drive the loss of some of these antimicrobial peptides that initiate a response towards gut microbiota thereby improving survival rates because of a generalized dampened response to pathogens? While we cannot answer these questions based on our exploration of genome assemblies, our results hint at key roles of AMPs in the gut and in response to pathogens.

By exploring the evolution of highly variable gene families that have undergone duplication and losses, our results highlight the importance of building species-specific databases for small proteins such as AMPs. This library will also help in gaining a deeper understanding of the complex interplay between pathogens and bats, which as the COVID-19 pandemic reminds us, is crucial to the survival of both humans and bats. Although the specific functions of defensins and cathelicidins in the immune response of bats are yet to be discovered, the HDPs retrieved here can help us gain valuable insights into their adaptations to pathogen tolerance. Just as host defense proteins are being investigated for several human diseases, the HDP libraries reported here can uncover new therapeutic avenues for combating infectious diseases that are decimating bat populations such as white nose syndrome.

## Data availability statement

The datasets presented in this study can be found in online repositories. The names of the repository/repositories and accession number(s) can be found in the article/**Supplementary Material.**

## Author contributions

DM-S, APC, LMD, DAR and FXC conceived this study. FXC designed the pipeline and generated the final database guided by DM-S, LMD, and DAR. GMH reconstructed the ultrametric tree. GMP collected an early database of bat AMPs under MM’s supervision. MCWL and KRM analyzed sequences from this early database. NS and AMB assembled transcriptomes. NSP supervised TE annotation and curation. MJ supervised phylogenetic analyses. All authors contributed to editing and proofreading this manuscript.
