# Supplementary material for: The evolution of antimicrobial peptides in Chiroptera
Source: Front Immunol. 2023 Sep 26;14:1250229. doi: 10.3389/fimmu.2023.1250229 (PMC10562630; doi:10.3389/fimmu.2023.1250229)
Supplement: Supplementary file 2 [file DataSheet_1.docx]

Supplementary Material

The evolution of antimicrobial peptides in Chiroptera

**Francisco X. Castellanos, Diana Moreno-Santillán^*^, Graham M. Hughes, Nicole S. Paulat, Nicolette Sipperly, Alexis M. Brown, Katherine R. Martin, Gregory M. Poterewicz, Marisa C.W. Lim, Amy L. Russell, Marianne S. Moore, Matthew G. Johnson, Angelique P. Corthals, David A. Ray^*^ and Liliana M. Dávalos^*^**

*** Correspondence:** Corresponding Authors: liliana.davalos@stonybrook.edu; david.4.ray@gmail.com; diana.moreno@berkeley.edu

# Supplementary Data

The code and files generated in this study are deposited in https://zenodo.org/record/8144377

# Supplementary Tables

All Supplementary Tables and their descriptions are included in the file Supplementary Tables.xlsx.

# Supplementary Figures

## Supplementary Figures

**
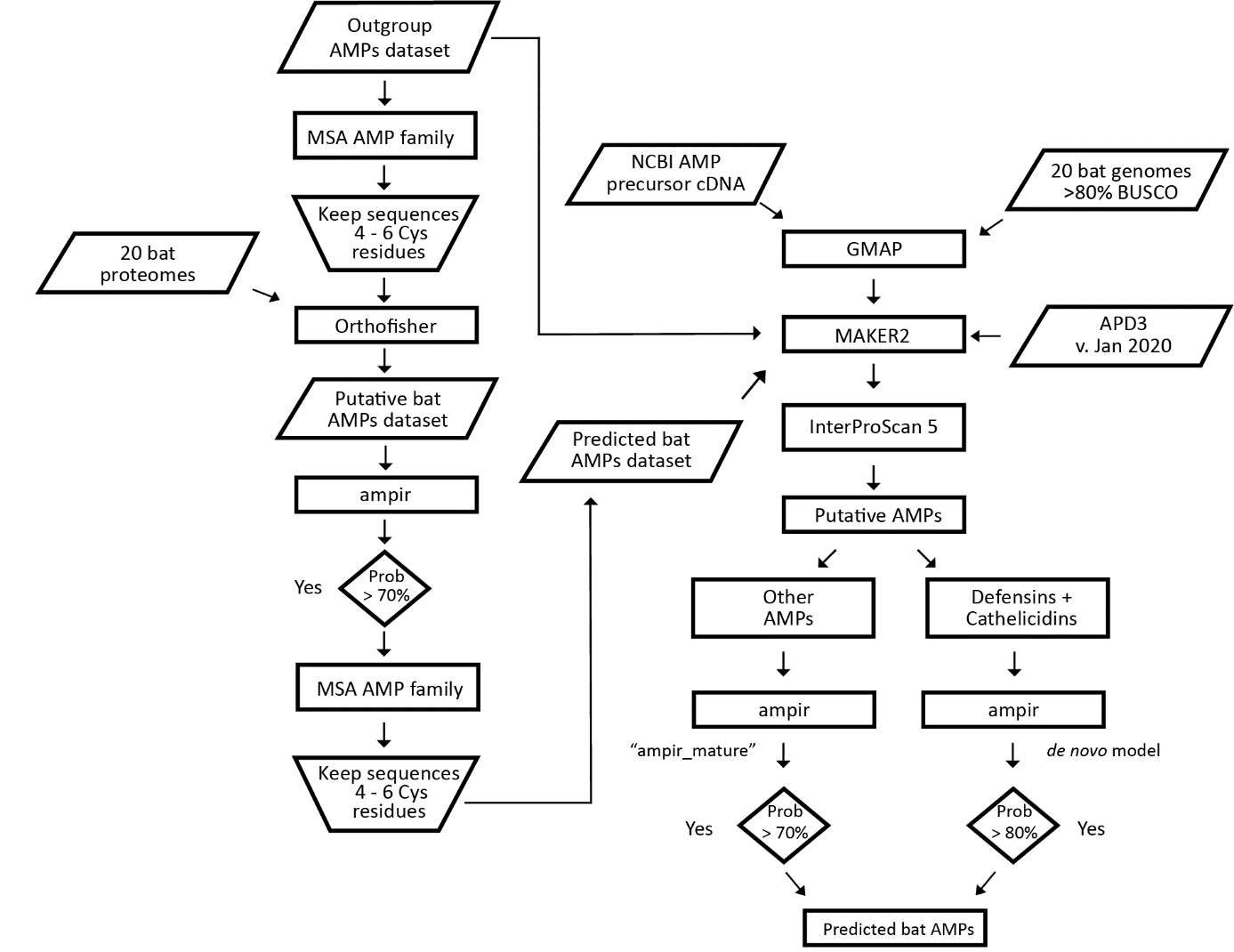
**

**Figure S1.** Summarized pipeline developed in this work to annotate and predict antimicrobial peptides in Chiroptera.


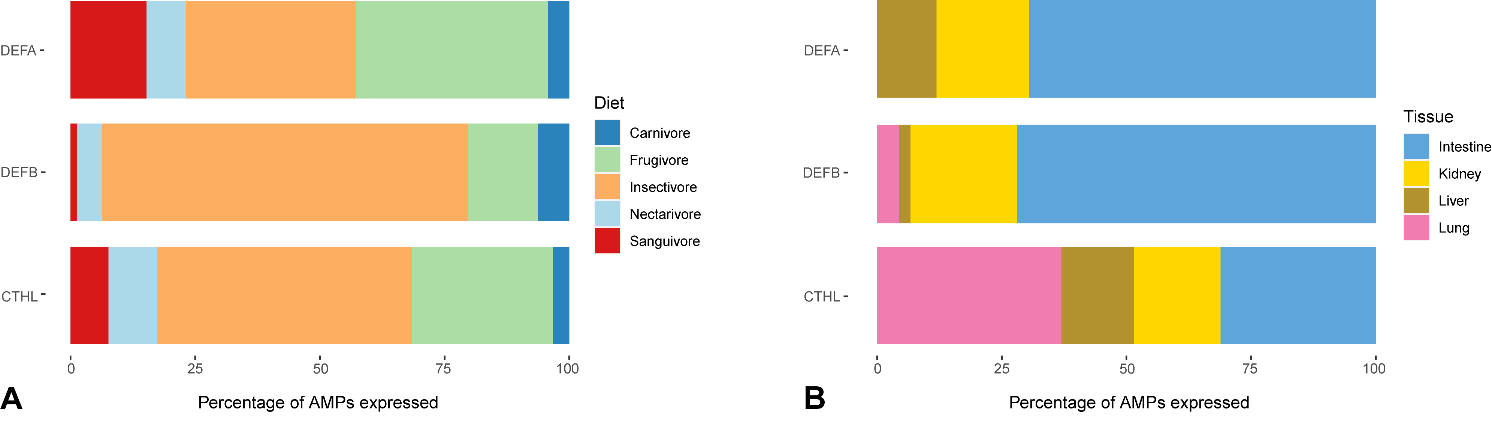


**Figure S2.** Expression analyses performed in published and *de novo* transcriptomic data based on bats’ feeding habits (A), and tissues examined (B).


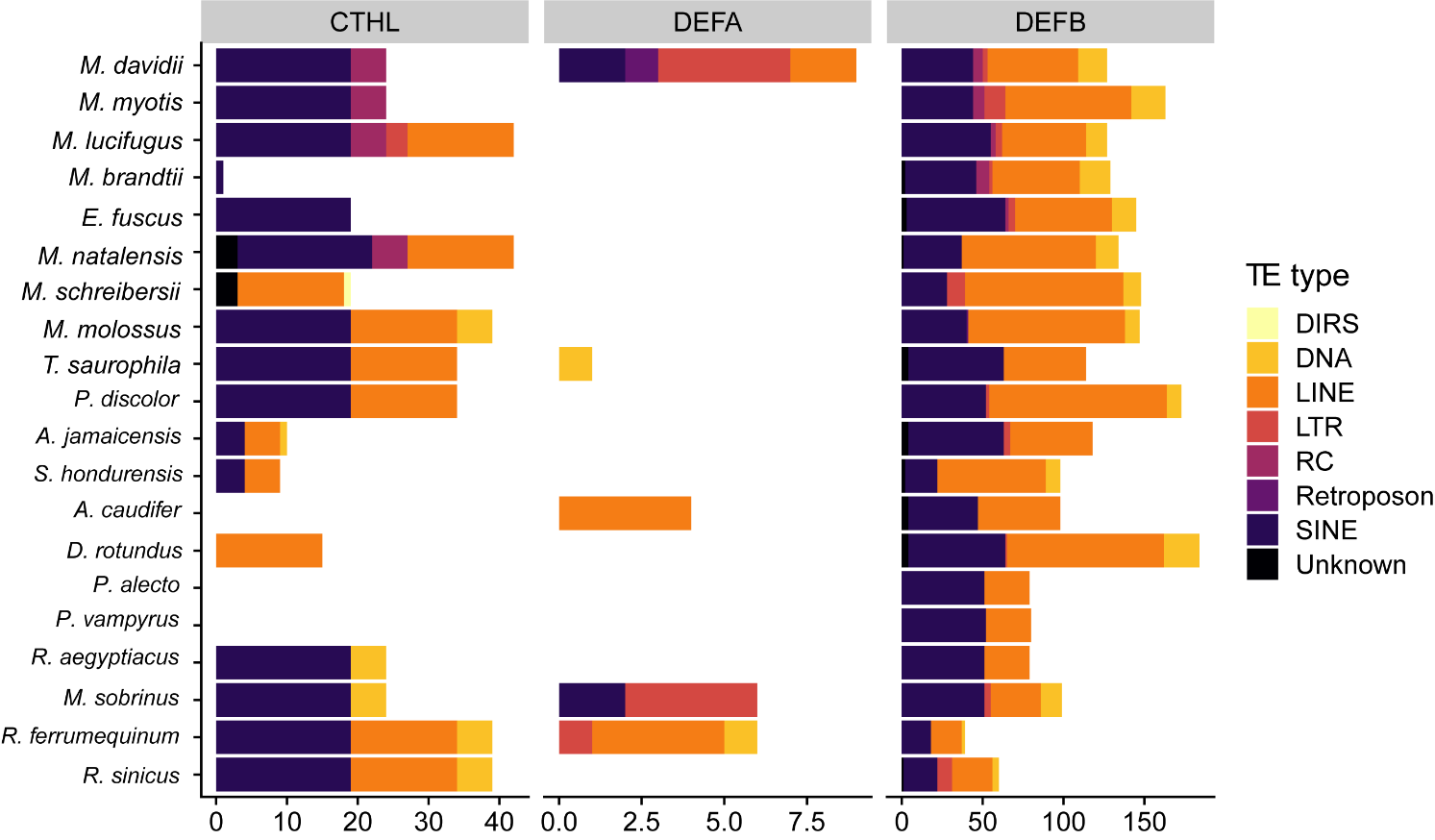


**Figure S3.** Stacked barplots displaying the differential TE type accumulation in some cathelicidins (CTHL), α-, β-defensins (DEFA, DEFB) gene introns.
